# Moment-to-moment affective dynamics in schizophrenia and bipolar disorder

**DOI:** 10.1192/j.eurpsy.2023.2438

**Published:** 2023-08-07

**Authors:** Suzanne Ho-wai So, Anson Kai Chun Chau, Lawrence Kin-hei Chung, Chung-ming Leung, George H.C. Chong, Wing Chung Chang, Arthur D.P. Mak, Sandra S.M. Chan, Sing Lee, Iris E. Sommer

**Affiliations:** 1 Department of Psychology, The Chinese University of Hong Kong, Hong Kong, China; 2 Institute of Health Equity, The Chinese University of Hong Kong, Hong Kong, China; 3 Department of Clinical Psychology, Kwai Chung Hospital, Hong Kong, China; 4 Department of Psychiatry, The University of Hong Kong, Hong Kong, China; 5 State Key Laboratory of Brain and Cognitive Sciences, The University of Hong Kong, Hong Kong, China; 6 Department of Psychiatry, The Chinese University of Hong Kong, Hong Kong, China; 7 Department of Psychiatry, University Medical Centre Groningen, The Netherlands

**Keywords:** affectivity, ecological momentary assessment, experience sampling, stress reactivity, transdiagnostic

## Abstract

**Background:**

Affective disturbances in schizophrenia and bipolar disorder may represent a transdiagnostic etiological process as well as a target of intervention. Hypotheses on similarities and differences in various parameters of affective dynamics (intensity, successive/acute changes, variability, and reactivity to stress) between the two disorders were tested.

**Methods:**

Experience sampling method was used to assess dynamics of positive and negative affect, 10 times a day over 6 consecutive days. Patients with schizophrenia (*n* = 46) and patients with bipolar disorder (*n* = 46) were compared against age-matched healthy controls (*n* = 46).

**Results:**

Compared to controls, the schizophrenia group had significantly more intense momentary negative affect, a lower likelihood of acute changes in positive affect, and reduced within-person variability of positive affect. The bipolar disorder group was not significantly different from either the schizophrenia group or the healthy control group on any affect indexes. Within the schizophrenia group, level of depression was associated with weaker reactivity to stress for negative affect. Within the bipolar disorder group, level of depression was associated with lower positive affect.

**Conclusions:**

Patients with schizophrenia endured a more stable and negative affective state than healthy individuals, and were less likely to be uplifted in response to happenings in daily life. There is little evidence that these affective constructs characterize the psychopathology of bipolar disorder; such investigation may have been limited by the heterogeneity within group. Our findings supported the clinical importance of assessing multiple facets of affective dynamics beyond the mean levels of intensity.

## Introduction

Schizophrenia and bipolar disorder, presented as distinct and severe mental disorders, share important similarities [1, 2]. There is a high level of comorbidity of psychotic and affective symptoms across both disorders [3–8]. Common DNA variants and brain changes that appear to influence the risk of both schizophrenia and bipolar disorder have been identified [9, 10], and an individual’s risk of suffering from either schizophrenia or bipolar disorder is elevated if a first-degree relative has one of the disorders [11], indicating a significant overlap in genetic factors underlying the two disorders [12]. Therefore, it would be of theoretical and clinical value to examine similarities and differences in psychological processes across disorders [13, 14].

Affective instability encompasses the following key dimensions: affective valence, affect amplitude/intensity, rapid affect shifting/oscillation, low reactivity threshold to environmental triggers, and the perceived capacity to control affect [15, 16]. While affective instability is most well-researched in conditions where it is a core symptom, such as borderline personality disorder [15, 17–19], it is frequently documented by clinicians of individuals with bipolar disorder, personality disorder, and schizophrenia [20]. Across disorders, implications of affective instability on clinical outcomes such as poor functioning and quality of life, prolonged hospitalization, increased use of healthcare services, and suicidality have been reported [20–24].

Abnormalities in affective dynamics, assessed by structured interviews or questionnaires such as Affective Lability Scale (ALS) [25] and Affective Intensity Measure (AIM) [26], are strongly implicated in schizophrenia and bipolar disorder. Despite the fact that schizophrenia-spectrum disorder is sometimes named “non-affective psychosis,” high negative affect and low positive affect are common among individuals with this diagnosis [22, 27]. It has been replicated that mood disturbances and instability predate and maintain psychotic symptoms [28–33]. Among individuals with liability to develop schizophrenia, it has been shown that individuals with negative schizotypy had reduced trait positive affect, whereas those with both negative and positive schizotypy had heightened trait negative affect and diminished emotional clarity [34]. Patients with bipolar disorder (both types I and II) are characterized by an increase in mood intensity and lability [35–39]. Notably, mood intensity and lability are evident even among euthymic or at-risk individuals [37, 40, 41].

While affective dynamics may represent a transdiagnostic risk factor for psychopathology in general, little evidence has specified the nuanced similarities and differences in patterns of these dynamics across specific forms of psychopathology [20, 42, 43]. This is especially important for severe mental disorders such as schizophrenia and bipolar disorder, where affective disturbances are common, complex, and persisting, and have been suggested as targets for treatment [37, 44]. In addition, it has been argued that research on the affectivity of schizophrenia has emphasized more on observed expressions than subjective affective experiences [45], and that of bipolar disorder has focused on mood periods rather than momentary affect [46]. Therefore, examination of various dimensions of affective instability across disorders using time-intensive methods such as experience sampling methodology (ESM) would help to advance this area of research [47–49].

ESM is a diary method that records the moment-by-moment context and subjective experiences in the flow of daily life [50–55]. ESM provides a prospective and ecologically valid representation of how experiences unfold and interact, avoiding retrospective recall bias. This is best-suited for assessing affective experiences, which are brief and can shift rapidly, as opposed to moods, which are more static and prolonged [56]. ESM questionnaires can provide a fine-grained record of the following parameters of affective dynamics within and between individuals and groups [49, 57–59]: (i) valence and intensity of affect, (ii) successive/acute changes in affect, (iii) overall variability of affect, (iv) affective reactivity to stress, and (v) affective modulation. Unlike major depressive disorder, where merely the mean level of negative affect is implicated [60], the affective dynamics of schizophrenia and bipolar disorder may be more complex; hence, comparisons on mean levels of positive affect (PA) and negative affect (NA) as well as the full range of affective instability subcomponents will be more informative in predicting clinical outcomes [19, 49, 61–63].

ESM studies in schizophrenia reported that patients experienced more intense and more variable NA, and less intense and less variable PA, than controls [45, 64, 65]. Among patients with schizophrenia, NA not only sustain each other (e.g., sadness leading to diminished happiness [66]) but also co-occur with PA, leading to difficulty in mood regulation [67–69]. In terms of affective reactivity to stress, patients with schizophrenia have been shown to report more NA (and less PA) following stressful daily-life events than healthy individuals [70–74]. On the other hand, they fail to maintain or increase the intensity of PA over time – a deficit that has been suggested to be associated with anhedonia [75].

Several ESM studies for bipolar disorder were conducted with non-clinic samples, where the psychometric risk for bipolar disorder was associated with more intense PA and NA, greater variability of NA, and greater affective reactivity to daily-life stress [76–78]. Variability and successive changes in NA predicted the subsequent emergence of bipolar spectrum disorders 3 years later [62]. The anecdotal ESM studies that involved patients with bipolar disorder reported higher NA and lower PA than healthy individuals, greater affect variability, and comparable affective reactivity to daily-life stress except for patients with depressive symptoms [79, 80]. So far, one ESM study [71] had compared the two clinical groups, which was on affective reactivity to stress only. They reported that while daily-life stress triggered an increase in NA and a decrease in PA in patients with psychosis, it only triggered a decrease in PA in the bipolar disorder group.

Using ESM that can provide unique time-sensitive information about affective dynamics not captured by questionnaires [81], the present study aimed to compare various parameters of affective instability in schizophrenia and bipolar disorder.

The key hypotheses are as follows:

1. Compared to healthy controls, the intensity of momentary NA will be higher in the two clinical groups, whereas the intensity of momentary PA will be lower in the schizophrenia group only
2. Compared to healthy controls, successive/acute changes and overall variability in NA will be greater in the two clinical groups, whereas successive/acute changes and overall variability in PA will be greater in the bipolar disorder group only
3. Compared to healthy controls, momentary stress will lead to reduced PA in both clinical groups, and increased NA in the schizophrenia group only

In view of the accumulating evidence that affective dynamics may be differentially associated with symptoms, especially among individuals with schizophrenia [82–86], we also explored the association between specific parameters of affective dynamics and severity of symptoms within the two clinical groups respectively.

## Methods

This project was approved by the Joint Chinese University of Hong Kong – New Territories East Clinical Ethics Committee (CRE-2013.652-T, 2015.685-T and 2020.477), the Institutional Review Board of the University of Hong Kong/Hospital Authority Hong Kong West Cluster (UW15–194), and the Survey and Behavioral Research Committee (SBRE-19-788). All participants provided written informed consent for study participation.

### Sample

The sample consisted of three groups of adults (age range: 18–64). The inclusion criterion of the schizophrenia group (“SCZ”) was a diagnosis of schizophrenia, whereas the inclusion criterion of the bipolar disorder group (“BD”) was a diagnosis of bipolar I or II disorder. Diagnoses were individually assessed by the Structured Clinical Interview for Diagnostic and Statistical Manual of Mental Disorders, fourth edition (SCID-IV) [87]. In order to obtain clearly defined diagnostic groups, individuals who had schizoaffective disorder or a comorbid schizophrenia and bipolar disorder were excluded from the study. Age-matched healthy controls (“HC”), who reported no psychiatric diagnoses (confirmed by SCID), were recruited. Exclusion criteria for all groups were intellectual disability, substance-induced psychosis, organic brain syndrome, and a history of brain injury.

The clinical sample was referred by out-patient psychiatric clinics, with suitability of study participation being confirmed by the care teams. Healthy individuals were recruited through a university subject pool, public channels including social media, and dissemination of leaflets. A proportion of the clinical sample was shared with previous studies [88–90], but the analysis of the present study was novel. All healthy controls were new.

For sample size calculation, G*power suggested a total sample size of 159 for 3-group comparisons with a medium effect size (*f* = 0.25, power = 0.80, alpha = 0.05). On the contrary, since multiple ESM observations are nested within an individual, Hox [91] suggested the “50/20 rule” for testing cross-level interactions of nested data, where a minimum of 20 ESM measurements nested within a minimum of 50 participants in total would be adequately powered for group differences in ESM variables.

### Measures

#### Experience sampling method

All participants were required to complete repeated measures using a mobile phone app developed by our research team [88]. The app was installed either on their own mobile phone or on an iPod Touch borrowed from our team. The app emitted a signal at 10 random moments during the day over 6 consecutive days. Upon each signal, participants were probed to answer the same set of questions that concerned their momentary affective experiences, contextual information, and perceived stress of the event that had happened since the last signal. The questionnaire was only active for 15 min after the signal, and successive signals were at least 30 min apart. Except for contextual information, all other ESM items were rated on 7-point Likert scales (1 = not at all; 7 = very). Only participants who completed one-third of the ESM entries were included in data analysis [92].

Three items on PA (happy, relaxed, contented) and three items on NA (irritated, low, nervous) were included. As these items have been shown to represent PA and NA reliably in previous studies [88, 93, 94], we reported *intensity* of momentary PA and NA by averaging the three respective items per time point [71]. In our sample, the within-person reliabilities of the PA index score and the NA index score were good (SCZ = 0.74, BD = 0.80, HC = 0.85) and satisfactory (SCZ = 0.68, BD = 0.61, HC = 0.73), respectively. The between-person reliabilities of the PA index score and the NA index score were excellent (SCZ = 0.98, BD = 0.97, HC = 0.96) and good (SCZ = 0.92, BD = 0.93, HC = 0.85), respectively [95].

Following Jahng et al. [57] and Sperry and Kwapil [96], *successive changes* and *acute changes* in PA and NA were calculated using the adjusted squared successive differences (ASSD) and probabilities of acute change (PAC), respectively. ASSD captures the moment-to-moment fluctuations in affect, taking into consideration the within-person variability and temporal dependency (i.e., autocorrelation). The formula for ASSD calculation is shown below [57, 96]:

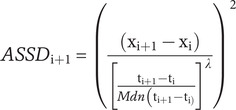



Lambda (λ) was computed using non-parametric smoothing regressions that minimize the sum of squares between successive differences, which were adjusted by the median (Mdn) of the time intervals. In the calculation of the successive differences, the first ESM entry of each day was treated as missing, where Mdn time for all intervals = 71.1 mins, λ for NA = 0.38 and λ for PA = 0.02.

PAC determines if the momentary fluctuation in affect is considered as large relative to the ASSD. Following Jahng et al. [57] and Sperry and Kwapil [96], ASSDs above the 90th percentile were considered as acute (coded as 1), and ASSDs below that cutoff were coded as 0.

*Overall variability* was represented by the within-person standard deviation in PA or NA item responses for each participant respectively [60]. *Affective reactivity to stress* was calculated based on Myin-Germeys et al. [71]. At each momentary questionnaire, participants were asked to identify the key activity between the current and preceding beeps, and to evaluate their subjective stress level with the question “How stressful was the activity?” [93]. Therefore, within-moment associations between activity stress and PA and NA could be understood as the lagged effect of stress (experienced between two moments) on affect at the current moment [71, 72].

#### Clinical rating scales

The schizophrenia group completed the Positive and Negative Syndrome Scale (PANSS) [97] and the Calgary Depression Scale for Schizophrenia (CDSS) [98], which measured the severity of psychotic and depressive symptoms, respectively. The bipolar disorder group completed the Young Mania Rating Scale (YMRS) [99] and the Montgomery-Asberg Depression Scale (MADRS) [100], which assessed levels of manic and depressive symptoms, respectively.

### Procedure

Upon informed consent, participants went through a structured interview, consisting of the SCID, PANSS, and CDSS (for SCZ), YMRS and MADRS (for BD), conducted by a trained and supervised graduate-level psychologist. After the interview, participants were guided through the ESM assessment so that they felt confident completing the subsequent assessment on their own. During the ESM assessment period, the experimenter contacted the participant occasionally to encourage assessment compliance and to provide technical support.

### Statistical analysis

Group differences in intensity, ASSD, and PAC of PA and NA were tested with Group as a categorical independent variable in the multilevel regression models [57], as ESM data involve momentary observations (level 1) nested within each participant (level 2) [101]. As PAC of each moment is a binary variable, group differences were tested using a logistical multilevel model. For overall variability, ANOVA tests were conducted to compare groups on within-person SD of PA and NA, respectively. For affective reactivity to stress, the association between level of stress and affect was estimated by multilevel regression models, with momentary stress level as independent variable and momentary PA and NA as outcome variables in separate models. Group comparisons in affective reactivity were examined by multilevel models where Group, Stress, and Group × Stress interaction terms were entered as independent variables, and their random effects modeled. Group differences in affective reactivity would be implicated by the statistical significance of the Group × Stress interaction term. Three-group comparisons were followed by pairwise comparisons on each variable.

As exploratory analyses, person-level correlation and regression were tested to examine associations between affective dynamics and severity of symptoms within each clinical group. All analyses were conducted with JAMOVI v 2.3.2.0 [102].

## Results

A total of 61 patients with schizophrenia and 65 patients with bipolar disorder were eligible and recruited for the study, among whom 46 (75.4%) and 46 (70.8%) met the ESM completion rate threshold, respectively. An equal number of healthy controls were randomly selected from our existing datasets (age and gender stratified), making a final sample of 138 participants (SCZ = 46, BD = 46, HC = 46) for data analysis.

### Sample characteristics

Demographic characteristics of the sample are presented in Table 1. The three groups were matched on age and gender (*ps* > 0.050). HC had a higher education level than the two clinical groups (SCZ vs HC: *t*(87.93) = 4.93, *p* < 0.001; BD vs HC: *t*(89.30) = 3.11, *p* = 0.002). The highest income category was most common in HC, whereas the lowest income category was most common in SCZ.

**Table 1.** Sample characteristics

	SCZ (*n* = 46)	BD (*n* = 46)	HC (*n* = 46)	Group comparisons
Age	41.48 (12.31)	39.00 (11.91)	37.41 (13.83)	*F*(2, 135) = 1.20, *p* = 0.306
Gender				Χ^2^(2) = 5.79, *p* = 0.055
Male	22 (47.8%)	11 (23.9%)	18 (39.1%)	
Female	24 (52.2%)	35 (76.1%)	28 (60.9%)	
Year of education	12.15 (3.36)	13.41 (3.15)	15.27 (2.88)	*F*(2, 135) = 12.31, *p* < 0.001
Household income	(*n* = 33)	(*n* = 45)	(*n* = 46)	Χ^2^ (6) = 27.37, *p* = <0.001
<HKD10,000	17 (51.5%)	13 (28.9%)	3 (6.5%)	
HKD10,000–29,999	9 (27.3%)	19 (42.2%)	15 (32.6%)	
HKD30,000–49,999	5 (15.2%)	8 (17.8%)	13 (28.3%)	
>HKD50,000	2 (6.1%)	5 (11.1%)	15 (32.6%)	

Abbreviations: BD, bipolar disorder; HC, healthy controls; SCZ, schizophrenia.

The SCZ group consisted of patients with a SCID diagnosis of schizophrenia (100%). The average duration of illness was 14.18 years (SD = 12.13, range = 1–44). Mean scores of clinical rating scales were as follows: PANSS positive score = 17.52 (SD = 6.70, range = 9–32), PANSS negative score = 10.72 (SD = 3.86, range = 7–23), PANSS general score = 25.09 (SD = 6.74, range = 16–45), PANSS total score = 53.33 (SD = 13.55, range = 33–83), CDSS total score = 3.24 (SD = 3.20, range = 0–12).

The BD group had an average duration of illness of 11.16 years (SD = 10.80, range = 0–40). The majority of the group (*n* = 33, 71.7%) had a diagnosis of bipolar I disorder, whereas 13 patients (28.3%) had bipolar II disorder. Eighteen patients (39.1%) were in an active episode at the time of assessment (mixed: *n* = 7, depressive: *n* = 3, manic/hypomanic: *n* = 8). Mean scores of clinical rating scales were as follows: YMRS = 2.16 (SD = 3.94, range = 0–15), MADRS = 6.77 (SD = 7.91, range = 0–29).

Diagnostic interviews revealed that none of the healthy controls reached the diagnostic threshold for any DSM-IV psychiatric disorders. As shown in Table 2, there was a significant group difference in ESM compliance, with HC completing more entries than SCZ (*t*(135) = −1.93, *p* = 0.056, *d* = 0.40) and BD (*t*(135) = −2.59, *p* = 0.011, *d* = 0.54).

**Table 2.** Indicators of affective dynamics across groups

	SCZ (*n* = 46)	BD (*n* = 46)	HC (*n* = 46)	Group comparisons	SCZ vs HC	BD vs HC	SCZ vs BD
ESM entry compliance	68.2% (0.20)	65.5% (0.20)	76.1% (0.19)	*F*(2, 135) = 3.62, *p* = 0.030, η^2^ = 0.05	*t*(135) = −1.93, *p* = 0.056, d = 0.40	*t*(135) = −2.59, *p* = 0.011, d = 0.54	*t*(135) = 0.66, *p* = 0.508, d = 0.14
Mean PA	4.21 (1.41)	4.37 (1.16)	4.65 (1.00)	*F*(2, 138.05) = 1.62, *p* = 0.201, η^2^ = 0.02	*t*(137.88) = −1.78, *p* = 0.077, *d* = 0.30	*t*(138.01) = −1.11, *p* = 0.267, *d* = 0.19	*t*(138.27) = −0.67, *p* = 0.505, *d* = 0.11
Mean NA	2.79 (1.17)	2.51 (1.07)	2.28 (0.82)	*F*(2, 138.06) = 2.89, *p* = 0.059, η^2^ = 0.04	*t*(137.86) = 2.40, *p* = 0.018, *d* = 0.41	*t*(128.01) = 1.10, *p* = 0.273, *d* = 0.19	*t*(138.32) = 1.30, *p* = 0.196, *d* = 0.22
Mean ASSD (PA)	1.01 (1.13)	1.03 (0.91)	1.29 (0.99)	*F*(2, 141.33) = 1.27, *p* = 0.285, η^2^ = 0.02	*t*(137.95) = −1.43, *p* = 0.155, *d* = 0.24	*t*(141.44) = −1.31, *p* = 0.192, *d* = 0.22	*t*(144.91) = −0.11, *p* = 0.914, *d* = 0.02
Mean ASSD (NA)	1.20 (1.35)	0.87 (0.86)	1.08 (0.86)	*F*(2, 143.76) = 1.06, *p* = 0.349, η^2^ = 0.01	*t*(139.87) = 0.40, *p* = 0.687, *d* = 0.07	*t*(143.83) = −1.02, *p* = 0.308, *d* = 0.17	*t*(147.98) = 1.41, *p* = 0.159, *d* = 0.23
Mean PAC (PA)	0.09 (0.14)	0.09 (0.11)	0.13 (0.12)	Χ^2^ (2) = 5.77, *p* = 0.056, V = 0.14	*Z* = −2.30, *p* = 0.021, OR = 0.47	*Z* = −1.66, *p* = 0.097, OR = 0.58	*Z* = −0.64, *p* = 0.521, OR = 1.25
Mean PAC (NA)	0.10 (0.12)	0.08 (0.10)	0.11 (0.11)	Χ^2^ (2) = 3.07, *p* = 0.216, V = 0.11	*Z* = −0.80, *p* = 0.424, OR = 0.78	*Z* = −1.75, *p* = 0.080, OR = 0.56	*Z* = 0.96, *p* = 0.336, OR = 0.73
Variability (PA)	0.77 (0.44)	0.84 (0.33)	0.98 (0.37)	*F*(2, 135) = 3.52, *p* = 0.032, η^2^ = 0.05	*t*(135) = −2.60, *p* = 0.010, *d* = 0.54	*t*(135) = −1.75, *p* = 0.082, *d* = 0.37	*t*(135) = −0.85, *p* = 0.397, *d* = 0.18
Variability (NA)	0.79 (0.46)	0.71 (0.34)	0.85 (0.35)	*F*(2, 135) = 1.55, *p* = 0.217, η^2^ = 0.03	*t*(135) = −0.67, *p* = 0.504, *d* = 0.14	*t*(135) = −1.74, *p* = 0.084, *d* = 0.36	*t*(135) = 1.07, *p* = 0.285, *d* = 0.22
Mean Event-related stress	2.73 (1.17)	2.46 (1.06)	3.05 (1.41)	*F*(2, 135.51) = 2.50, *p* = 0.086, η^2^ = 0.02	*t*(135.68) = 1.15, *p* = 0.253, *d* = 0.20	*t*(135.95) = −2.23, *p* = 0.027, *d* = 0.38	*t*(134.89) = 1.09, *p* = 0.278, *d* = 0.19

Abbreviations: BD, bipolar disorder; HC, healthy controls; NA, negative affect; PA, positive affect; SCZ, schizophrenia.

### Group comparisons of intensity of momentary affect (Hypothesis 1)

There was a marginally significant 3-group difference in NA (*F*(2, 138.06) = 2.89, *p* = 0.059, η^2^=0.04). Pairwise comparisons revealed that the level of NA was significantly higher in SCZ than HC (*t*(137.86) = 2.40, *p* = 0.018, *d* = 0.41). There was no significant difference in NA between BD and HC, or between SCZ and BD (*ps* > 0.050).

The momentary level of PA was not significantly different across groups (*p* > 0.050).

### Group comparisons of instability and overall variability of affect (Hypothesis 2)

There was a marginally significant three-group difference in PAC of PA (*Χ* ^2^(2) = 5.77, *p* = 0.056, *V* = 0.14). Pairwise comparisons revealed that PAC of PA was significantly lower in SCZ than HC (*Z* = -2.30, *p* = 0.021, OR = 0.47). There was no significant difference in PAC of PA between BD and HC, or between SCZ and BD (*ps* > 0.050). The mean ASSDs of PA and NA, and the mean PAC of NA were not significantly different across groups (*ps* > 0.050).

There was a significant three-group difference in variability of PA (*F*(2, 135) = 3.52, *p* = 0.032, η^2^=0.05). Pairwise comparisons revealed that variability of PA was significantly lower in SCZ than HC (*t*(135) = −2.60, *p* = 0.010, *d* = 0.54). Variability of NA was not significantly different across groups (*p* > 0.050).

### Group comparisons of affective reactivity to stress (Hypothesis 3)

As shown in Table 2, there was no overall group difference in momentary level of event-related stress (*p* > 0.050).

Across the entire sample, momentary level of event-related stress was positively associated with NA (*B* = 0.25, *p* < 0.001) and negatively associated with PA (*B* = -0.18, *p* < 0.001). The effects of Group × Stress interaction on either PA (*F*(2, 134.38) = 0.44, *p* = 0.643, η^2^=0.01) or NA (*F*(2, 133.24) = 1.44, *p* = 0.241, η^2^=0.02) were not significant, indicating no group difference in affective reactivity to stress.

**Figure 1.**
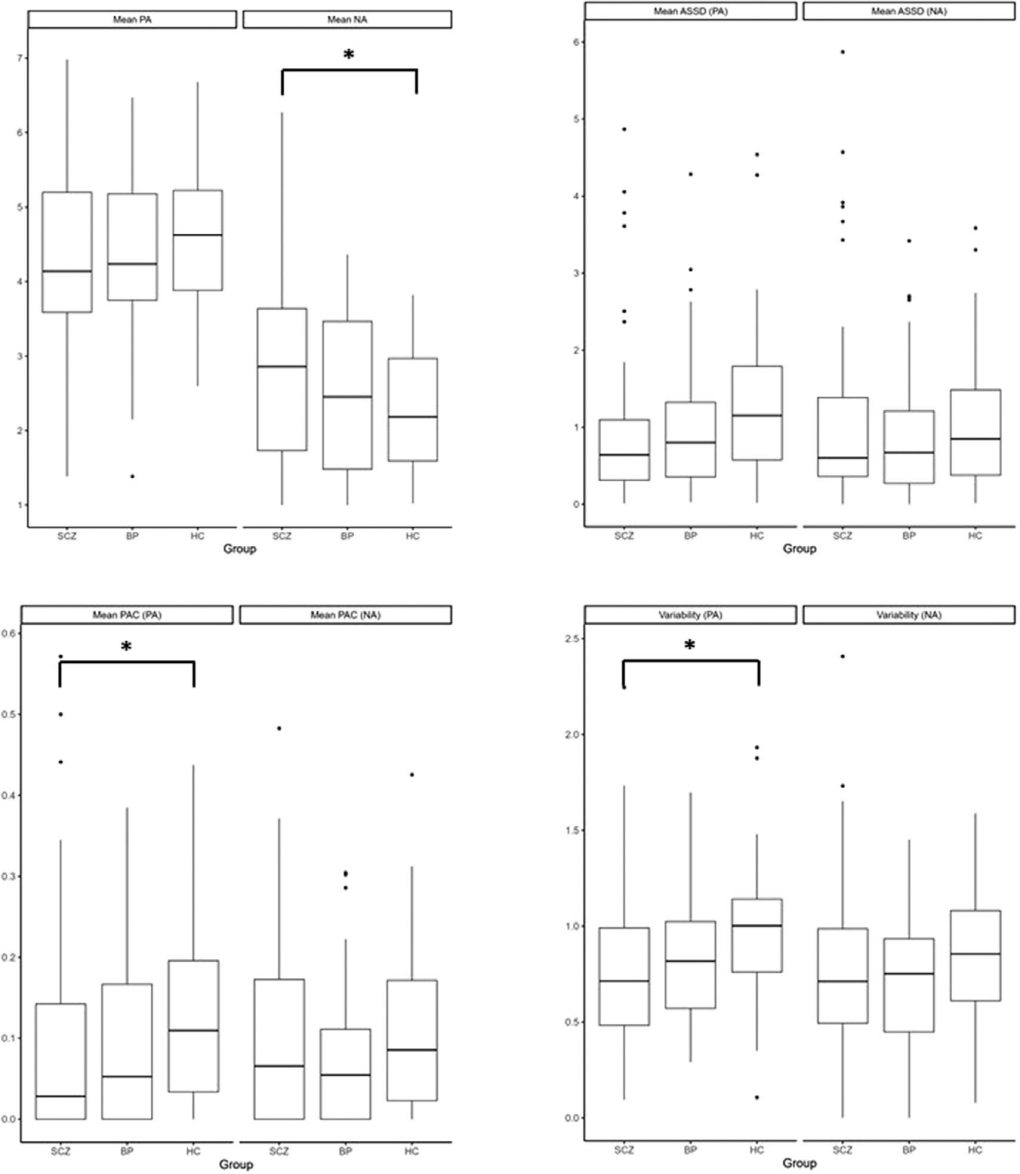
Box plots of affective dynamics across groups. *p < 0.050. BP, bipolar disorder; HC, healthy controls; SCZ, schizophrenia.

**Figure 2.**
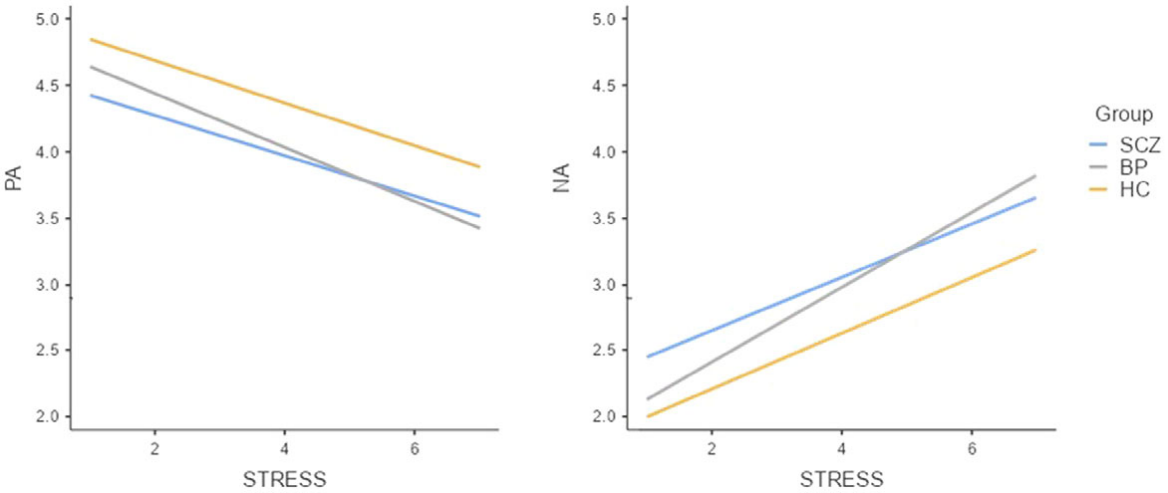
Affective reactivity to stress for PA (left) and NA (right) across groups. BP, bipolar disorder; HC, healthy controls; SCZ, schizophrenia.

Group comparisons on all affective dynamics are summarized in Figures 1 and 2.

### Exploratory analyses: Relationships between affective dynamics and symptom severity

Within the SCZ group, CDSS total score was associated with weaker affective reactivity to stress for NA (stress × CDSS total score interaction: B = -0.02, *p* = 0.024). PANSS total score and subscores were not significantly correlated with any of the affective indexes (*ps* > 0.050).

Within the BD group, the MADRS total score and intensity of PA were negatively correlated (rho = −0.44, *p* = 0.003). Patients diagnosed with Type I versus Type II BD did not differ on any of the affective dynamics (*p* > 0.050). On the other hand, compared with patients who were not in an active episode (*n* = 28), patients who were in an active episode (*n* = 18) had a lower intensity of PA (*t*(46.17) = −2.79, *p* = 0.008, *d* = 0.82), a higher intensity of NA (*t*(45.95) =2.65, *p* = 0.011, *d* = 0.78), and greater PAC for PA (*Z* = 1.97, *p* = 0.049, OR = 2.33).

## Discussion

This study compared moment-to-moment affective dynamics between patients with schizophrenia, patients with bipolar disorder, and healthy controls. Using subjective reports on the ESM, various components of affective intensity and instability were operationalized for both PA and NA. This allowed a systematic approach to examining the construct across disorders in context [44, 62].

Except for affective reactivity to stress, the other three aspects of affective dynamics (intensity, successive/acute changes, and variability) were different across groups. It is noteworthy that these overall differences were mainly driven by differences between the schizophrenia group and healthy controls.

Consistent with previous studies [45, 64, 65], we found a higher momentary level of NA in the schizophrenia group than controls, even though only one patient in this group was in a major depressive episode. This is an important finding because schizophrenia is sometimes translated as “non-affective psychosis,” denying the prominent presence of affective disturbances [22, 27]. In terms of fluctuations in affect, while successive changes of either PA or NA (represented by ASSD) were not different across groups, a lower PAC was observed in the schizophrenia group than controls. That is, patients were approximately half as likely (OR = 0.47) to experience a large acute fluctuation in PA compared to healthy individuals. In addition, within-person variability of PA was reduced in the schizophrenia group than controls. Recently, there has been considerable debate about the extent to which measures of affective dynamics provide additional information above and beyond mean levels of affect [60]. The multi-faceted differences between patients and controls in the present study suggested that a comprehensive assessment of affective dynamics beyond mean levels of affect may provide clinically relevant information.

Our finding that the schizophrenia group endured a more stable and negative affective state than healthy individuals, which are less likely to be uplifted in response to happenings in daily life, is consistent with Strauss et al.’s [75] suggestion that patients with schizophrenia are characterized with a deficit in augmenting positive affect. According to Scheffer et al. [103, 104], stronger density and inertia of negative affect are features of a system that is slow to recover from minor perturbations, leading to transitions into negative outcomes.

While the overall scores of the bipolar disorder group fell between the two other groups, there was no significant pairwise difference with either the schizophrenia group or the healthy control group. Post-hoc analyses within the bipolar disorder group revealed nuances in affective dynamics between patients of different episodic statuses. Specifically, patients who were in an active episode were more negative (and less positive) in affect, and had more acute changes in positive affect than those who were not in an active episode. Therefore, the lack of group differences against healthy controls could possibly be attributed to the fact that only a minority of patients in the bipolar disorder group were in an active episode. However, limited by small subgroups and imbalanced group sizes, we did not conduct formal comparisons between bipolar disorder subgroups and healthy controls. This is something that can be further visited by future research.

It is of note that the results for PA and NA were not entirely consistent, where group differences were not significant for the level of PA and fluctuations of NA. This suggests that the affective dynamics for PA and NA should be considered as separate constructs rather than direct opposites to each other, and hence assessing both PA and NA would be more informative. This also informs clinical care, as regulation of both PA and NA warrants therapeutic attention [105].

For both patients and controls, daily-life stress led to more NA and less PA in the next moment. The level of affective reactivity to stress did not significantly differ across groups. Although the clinical groups were symptomatic, including some who were in an active episode, their subjective levels of stress were not higher than healthy individuals. A possible explanation is that patients tend to engage in fewer activities, which may contribute to the narrow score range of stress and hence limits the effect of stress on affect. Another possibility is that patients are not more sensitive to daily stressors. Muddle et al.’s [106] meta-analysis on stress reactivity between patients and controls reported only a small difference and marked heterogeneity. Therefore, affective reactivity to stress in schizophrenia and bipolar disorder remains a topic for further research.

Our exploratory correlation analysis between clinical symptoms and affective dynamics did not lend support to the recent proposition that positive symptoms are related to increased intensity and frequency of affect change whereas negative symptoms are related to increased resistance to change from baseline [85]. It is of note that recent studies that found differential relationships between symptom profiles and specific aspects of affective dynamics tended to have larger samples of patients [82] and non-patients [83, 84]. In our study, within both clinical groups alike, severity of depressive symptoms was correlated with affective dynamics. The findings that depression was associated with lower PA (in the BD group) and lower NA reactivity to stress (in the SCZ group) may reflect a state of inertia.

This study has certain limitations. Informed by previous research, we analyzed PA and NA as groups of affective states. Separating the specific affective states, albeit leading to multiple comparisons, may result in a more complete representation of affective dynamics because variability in anxiety, anger, and sadness could potentially be distinct from each other [107]. In addition, in view of reports that psychiatric patients may display difficulties in identifying and labelling emotions [101, 108], the use of physiological assessment of affective experiences may supplement the subjective report and provide a more comprehensive understanding of affective dynamics. Lastly, as different clinical rating scales were selected to measure symptom severity for the two clinical groups respectively, the exploratory analyses on the association between symptom severity and affective dynamics could only be conducted within group.

In summary, the present study showed that patients with schizophrenia have elevated NA and reduced variability of PA. Addressing affective dynamic subcomponents across both positive and negative affect may inform illness management and affective regulation efforts.
